# Expression of α-Tubulin Acetyltransferase 1 and Tubulin Acetylation as Selective Forces in Cell Competition

**DOI:** 10.3390/cells10020390

**Published:** 2021-02-14

**Authors:** Amir Mohammad Rahimi, Mingfang Cai, Irem Kılıҫ, Zahra Basir Kazerouni, Constanza Tapia Contreras, Sigrid Hoyer-Fender

**Affiliations:** 1 GZMB, Johann-Friedrich-Blumenbach-Institute of Zoology and Anthropology—Developmental Biology, Biological Faculty, Georg-August-Universität Göttingen, Ernst-Caspari-Haus, Justus-von-Liebig-Weg 11, 37077 Göttingen, Germany; a.rahimi@stud.uni-goettingen.de (A.M.R.); mingfang.cai@stud.uni-goettingen.de (M.C.); irem.kilic46@gmail.com (I.K.); z.basirkazerouni@stud.uni-goettingen.de (Z.B.K.); Constanza.tapia-contreras@biologie.uni-goettingen.de (C.T.C.); 2 Department of Biology, School of Mathematics, Physics and Natural Sciences, University of Rome Tor Vergata, Via della Ricerca Scientifica, 1-00133 Rome, Italy

**Keywords:** fibroblasts, primary cilia, tubulin acetylation, *Atat1* expression, cultivation conditions, cell competition

## Abstract

The wound healing response of fibroblasts critically depends on the primary cilium, a sensory organelle protruding into the environment and comprising a stable axonemal structure. A characteristic marker for primary cilia is acetylation of axonemal tubulin. Although formation of primary cilia is under cell cycle control, the environmental cues affecting ciliation are not fully understood. Our purpose was, therefore, to study the impact of culture conditions on cilia formation in NIH3T3 fibroblasts. We quantified ciliation in different NIH3T3 sub-cell lines and culture conditions by immunodetection of primary cilia and counting. Quantitative Western blotting, qRT-PCR, and proliferation assays completed our investigation. We observed large differences between NIH3T3 sub-cell lines in their ability to generate acetylated primary cilia that correlated with cytoplasmic tubulin acetylation. We found no increased activity of the major tubulin deacetylase, HDAC6, but instead reduced expression of the α-tubulin acetyltransferase 1 (*Atat1*) as being causative. Our observations demonstrate that cells with reduced expression of *Atat1* and tubulin acetylation proliferate faster, eventually displacing all other cells in the population. Expression of *Atat1* and tubulin acetylation are therefore selective forces in cell competition.

## 1. Introduction

Fibroblasts are fundamental cells of the connective tissue. They maintain the structural integrity of the stroma by synthesizing the extracellular matrix. Furthermore, fibroblasts play a critical role in wound healing and inflammatory diseases, like rheumatism, and fibrotic diseases [1]. However, injury-stimulated migration of fibroblasts and their wound-healing capacity critically depend on a functional primary cilium [2]. In the initial phase of wound healing, activated platelets trigger the release of growth factors, such as platelet-derived growth factor (PDGF), that signal through the tyrosine kinase receptor PDGFRα located in the primary cilium membrane of fibroblasts causing re-entry into the cell cycle from quiescence and proliferation [3].

Primary cilia are solitary organelles that emerge from the cell surface into the environment. They consist of a centrally located axoneme surrounded by a specialized ciliary membrane and are generally immotile [4,5]. Primary cilia are present on nearly all cell types of the vertebrate body. They are essential sensory organelles transducing mechanical and chemical signals towards the cell center [4,6,7]. Therefore, they are crucial not only for embryonic and postnatal development, but also for tissue homeostasis in adulthood. Dysfunction of primary cilia causes severe human diseases collectively named ciliopathies, inter alia kidney diseases, neural tube defects, and left–right asymmetry [8,9,10,11,12,13,14,15,16,17]. Primary cilia are mainly found on G0-phase arrested cells that, eventually, spawned the current view that a primary cilium is built when the cell leaves the cell cycle to become quiescent and disintegrates when re-entering the cell cycle. In cell culture, a medium with reduced serum concentration, so-called serum starvation medium, is generally used to induce quiescence and the formation of primary cilia [18,19,20,21,22,23,24,25]. Beyond the regulation of ciliation by cell cycle exit, the disassembly of primary cilia seems to directly regulate G1/S transition indicating primary cilia as cell cycle regulators [24,26,27,28]. In this regard, the lower incidence or even absence of primary cilia in cancer cells seems to directly affect cell cycle progression but is not caused by ongoing proliferation [29,30].

A widely used and acknowledged marker for the detection of cilia is the acetylation of tubulin by routinely using the specific monoclonal antibody clone 6-11B-1 [31,32,33,34,35]. Although microtubules are subjected to diverse post-translational modifications, tubulin acetylation is the most prominent. Acetylated tubulin is present in long-lived stable structures as, e.g., centrioles and cilia. Furthermore, tubulin acetylation plays an important role in several cellular activities amongst them vesicle transport, signal transduction, and cell migration [36]. The transition of epithelial cells into mesenchymal cells (EMT), as observed in tumor formation but also wound healing and fibrosis, is characterized, inter alia, by an increased migratory behavior and a decrease in tubulin acetylation [37].

Acetylation of tubulin occurs mainly at lysine-40 (K40) of α-tubulin by the α-tubulin acetyltransferase 1 (ATAT1), which is also the major tubulin acetyltransferase in mammals. Modification of α-tubulinK40ac occurs on polymerized microtubules and is predominantly found on stable microtubules (MTs), whereas dynamic MTs miss this modification. Although cilia are predominantly characterized by acetylated tubulin, its impact on cilia formation, stability, and function is not fully understood. Due to the fact that tubulin acetylation seems to be important for cargo transportation, a conceivable scenario is its involvement in intraflagellar transport that is essential for ciliary assembly and disassembly [38,39,40]. However, in contrast to ciliation, tubulin acetylation is not essential for animal survival. Deletion of the mouse *Atat1* gene leads to complete loss of tubulin acetylation in embryos and a variety of tissues but animals display only mild phenotypes [41,42]. Furthermore, cilia are functional in *Atat1^-/-^* mice, and embryonic fibroblasts show only mild effects on proliferation and primary cilia formation [43,44]. Thus, acetylation of α-tubulin might rather be a marker of microtubule age conferring resilience against mechanical stress and aging [38]. In contrast, in vitro studies have demonstrated a correlation between tubulin deacetylation and ciliary disassembly, provoked by the histone deacetylase 6 (HDAC6), which is activated by Aurora kinase A-mediated phosphorylation [45]. These findings, suggesting that HDAC6-mediated tubulin deacetylation caused destabilization of MTs and ciliary disassembly, are contradictory to the observation that tubulin acetylation did not stabilize MTs [46,47]. Instead, acetylation takes place after stabilization and does not protect against depolymerization, but most likely enhances flexibility and robustness towards mechanical stress [34,47,48]. Additionally, irrespective of its acetyltransferase activity, the interaction of ATAT1 with MTs promotes MT destabilization and increases MT dynamics indicating that not acetylation but binding of ATAT1 is the critical factor regulating MT stability [49]. An exciting new facet of ATAT1 is the observation that ATAT1 controls cell growth via inhibition of the kinase AKT [50]. However, it is currently unknown whether the acetyltransferase activity and the growth controlling function of ATAT1 are interconnected.

Although the correlation between the presence of primary cilia and the cell cycle has been known for quite some time, its interdependency awaits further exploration [19,22,51]. Additionally, it is not known whether ciliogenic competence changes over time. We used here the mouse embryonic fibroblast cell line NIH3T3 as a model system to investigate the effect of culture conditions on the expression of primary cilia and more specifically on their tubulin acetylation. Cell cycle exit was induced by serum starvation (i.e., medium with reduced serum concentration) in a variety of NIH3T3 sub-cell lines, and ciliation investigated by immune detection of acetylated tubulin. Furthermore, the interrelationship between the cell cycle, the formation of primary cilia, and the acetylation of α-tubulinK40 was investigated by counting of primary cilia, quantitative Western blotting, qRT-PCR, and proliferation assays. We observed large differences between sub-cell lines in the amount of acetylated primary cilia that is caused by a decline in *Atat1* expression and tubulin acetylation. Furthermore, we demonstrate that long-term cultivation promoted the transformation of the cell population by cell competition in favor of cells with reduced *Atat1* expression and tubulin acetylation.

## 2. Materials and Methods

### 2.1. Cell Culture and Immunocytochemistry

NIH3T3 cells were obtained from ATCC (CRL-1658) (American Type Culture Collection, Manassas, Virginia, USA) and DSMZ (ACC59). Cells were routinely used for quite some time before and stored in liquid nitrogen with the exception of the newly obtained cell line from DSMZ (Deutsche Sammlung von Mikroorganismen und Zellkulturen, Braunschweig, Germany) named here NIH3T3 sub-cell line “DSMZ”. For all experiments, the sub-cell lines were freshly taken into culture. Cells were propagated in Dulbecco’s Modified Eagle’s Medium (DMEM; GlutaMax^TM^ with high glucose concentration (4.5 g/L); Thermo Fisher Scientific, Waltham, MA, USA, #10566), supplemented with 10% (*v*/*v*) fetal calf serum (FCS), 1000 U/mL penicillin, and 1000 µg/mL streptomycin at 37 °C and 5% CO_2_ (here consistently named as normal or standard medium), or supplemented with 0.5% FCS (serum starvation medium) [52]. For the induction of primary cilia, either serum starvation medium or old/exhausted medium was used. Old or exhausted medium was the supernatant of NIH3T3 cells that have proliferated in standard medium for ~3 days until confluency. The supernatant was aspirated and sterile filtered using 0.2 µm filters (Minisart, #16534-K; Sartorius, Göttingen, Germany) to remove any cellular components. HDAC6 was inhibited by the addition of 1.3 µM TSA (Trichostatin A, #19-138; Upstate Biotechnology—Fisher Scientific, Waltham, MA, USA), and SIRT2 by the addition of 5 mM nicotinamide (#72340; Sigma-Aldrich, St. Louis, Missouri, USA) for 24 h [47,53,54].

For immune cytology, cells were grown on glass coverslips in 6-well plates. They were fixed in 3.7% paraformaldehyde (PFA) for 20 min at 4 °C and permeabilized with 0.3% Triton X-100 in PBS (phosphate-buffered saline) for 10 min at room temperature. After rinsing in PBS, non-specific binding sites were blocked by incubation in PBS containing 1% bovine serum albumin (BSA) and 0.5% Tween-20 for at least 1 h. Samples were incubated with the primary antibodies anti-acetylated α-tubulin (clone 6-11B-1, #sc-23950, diluted 1:100; Santa Cruz Biotechnology, Inc., Santa Cruz, CA, USA), anti-Arl13b (#17711-1-AP, diluted 1:400; Proteintech Germany, St. Leon-Rot, Germany), anti-ODF2 (ESAP15572, diluted 1:100; antibodies-online, Aachen, Germany [55], anti-α-tubulin (#Ab-1, diluted 1:100; Oncogene, Merck Millipore, Burlington, MA, USA), or pAKT (Phospho-AKT (S473p) [587F11], Cell Signaling Technology, Danvers, MA, USA) at 4 °C overnight. Secondary antibodies used are goat-anti-mouse-IgG-DyLight 488 (#35503, Thermo Fisher Scientific, Waltham, MA, USA), goat-anti-mouse-IgG-AlexaFluor555 (#A21422, Lot 948498, Invitrogen; Invitrogen—Thermo Fisher Scientific, Waltham, MA, USA; and Molecular Probes, Eugene, Oregon, USA), and goat-anti-rabbit-MFP590 (#MFP-A1037, Mobitec, Göttingen, Germany), all diluted 1:1000. DNA was counterstained with DAPI. Images were taken by confocal microscopy (LSM 780, Carl Zeiss AG, Oberkochen, Germany) and processed using Adobe Photoshop 7.0. Primary cilia were manually counted by visual inspection and scanning through all focal planes. Approximately 500 cells for each replicate were scored for the presence of a primary cilium. The total counts are given in the results as n, comprising all replicates.

### 2.2. Protein Extraction and Western Blotting

Cells were trypsinized, counted, and washed 3× in PBS. Cells were lysed either by boiling immediately in 1× SDS-sample buffer or by incubation first in RIPA buffer (containing 1% NP-40, 1% Triton X-100, protease inhibitors) for 15 min followed by protein denaturation in SDS sample buffer. Protein lysates were separated on denaturing polyacrylamide gels [56], transferred to Hybond ECL membrane (Amersham Hybond-ECL, GE Healthcare, Chicago, Illinois, USA) [57], and incubated with the primary antibodies mouse anti-acetylated α-tubulin (6-11B-1, sc23950; Santa Cruz Biotechnology, Inc., Santa Cruz, CA, USA) and rabbit anti-ß-actin (ab8227, Abcam, Cambridge, Great Britain). Detection of primary antibodies was achieved by incubation with the fluorescent-labelled secondary antibodies IRDye800CW goat-anti-mouse IgG (#925-32210, LI-COR Biosciences GmbH, Bad Homburg, Germany) and IRDye680RD goat-anti-rabbit IgG (#925-68071, LI-COR Biosciences GmbH, Bad Homburg, Germany). Images were captured with LI-COR Odyssey. Images were quantified using Image Studio Lite (LI-COR Biosciences GmbH, Bad Homburg, Germany). The relative quantity of acetylated tubulin was calculated using the quantity of ß-actin in the same lane as reference. The fold changes of tubulin acetylation were calculated using the mean of the relative amount of acetylated tubulin in the standard medium as the reference.

### 2.3. Quantitative Reverse-Transcribed PCR (qRT-PCR)

Total RNA was prepared using peqGOLD RNApure™ (PeqLab, Erlangen, Germany) following the recommendations of the manufacturer, and digested with Ambion^®^ TURBO DNA-free™ DNase (Life Technologies, Carlsbad, CA, USA). cDNA was synthesized using Maxima First Strand cDNA Synthesis (Thermo Fisher Scientific, Waltham, MA, USA). The quantitative real-time PCR was performed on CFX96TM Real-Time System (Bio-Rad Laboratories, Inc., Hercules, CA, USA) using EvaGreen (Solis BioDyne, Tartu, Estonia). The following primers were used for expression analyses: *Hprt* (mHPRT-for2 ggagtcctgttgatgttgcc/mHPRT-rev2 gggacgcagcaactgacatt), *Gapdh* (mGapdhf CACCACCAACTGCTTAGCC/mGapdhr CGGATACATTGGGGGTAGG), and alpha-tubulin acetyltransferase1 (*Atat1*) (Atat1-for8 gcaggagacacacagactcc/Atata1-rev8 ctacaccctgggcatggaag). First, primer efficiency was validated for all primer pairs. The specificity of the amplification reaction was verified by melting curve analyses. Measurements were done in triplicates. The relative expression was calculated by ΔΔCt. Fold changes were calculated related to the mean expression of the housekeeping genes.

### 2.4. Proliferation Assay

Cells were seeded into 12-well plates using a defined cell concentration. At the indicated time points, triplicate wells were used, fixing the cells with 3.7% paraformaldehyde and staining with DAPI. Twenty pictures per replicate, all of a constant area, were captured using an inverted microscope (Zeiss Observer Z1, Carl Zeiss, AG, Oberkochen, Germany) and Image-Pro Plus 7.0. Nuclei were counted automatically and the increase in cell number calculated as log2. The proliferation rate in hours is calculated as the inverted slope.

### 2.5. Statistical Analyses

Data were analyzed and presented using Excel. The box in the boxplots represents the 25–75th percentile. The median is given as a line, the mean by a cross. The whiskers show the minimum and maximum values inside the range given by Q1-1.5x interquartile range (IQR) and Q3 + 1.5xIQR. Data were analyzed for normal distribution and by Student’s *t*-test.

## 3. Results

### 3.1. Cell Culture Conditions to Induce Primary Cilia Formation

**Figure 1:**
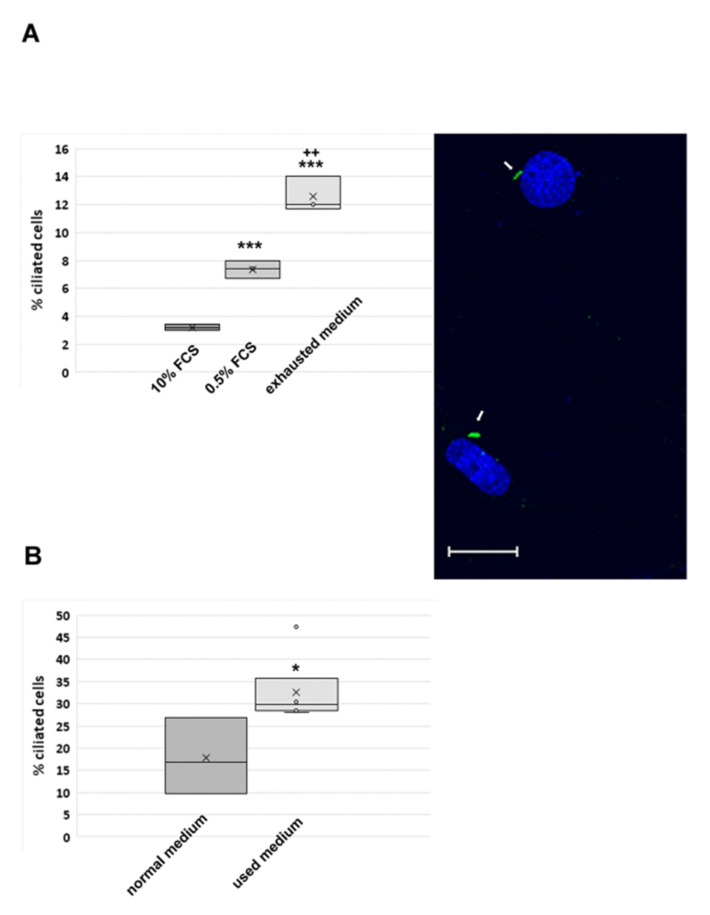
Induction of primary cilia in NIH3T3 cells by cultivation in the serum starved or exhausted (used) medium. (**A**) NIH3T3 cells (charge I) were seeded at a density of 2 × 10^5^ cells per 6-well plate and cultivated for 3 days in the specified medium. Primary cilia were decorated by acetyl-tubulin detection and counted. Statistically significant increase of ciliated cells in serum starved medium compared to cultivation in medium with 10% FCS (*p* < 0.001 ***), and between cultivation in serum starved medium (0.5% FCS) and exhausted medium (*p* < 0.01 ++). Triplicate experiments. Primary cilia decorated by acetylated tubulin staining (green, arrows) are exemplified (bar: 20 µm). (**B**) Primary cilia are already induced when NIH3T3 cells were cultivated in the exhausted (used) medium for only 18 h. 0.5 × 10^5^ cells (NIH3T3 charge II) were seeded into 6-well cell culture plates and cultivated in the normal or exhausted medium for up to 18 h. Primary cilia were detected by the decoration of acetylated tubulin and the percentage of ciliated cells determined. Triplicate experiment (normal medium *n* = 1563 cells; used medium *n* = 3100 cells counted). Statistically significant increase of ciliated cells in the used medium by Student’s *t*-test (*p* < 0.05 *; one-tailed, heteroskedastic). The median is represented as a line, the average as a cross.

We first verified whether our culture conditions are sufficient to induce cell cycle exit and the formation of primary cilia. To this end, primary cilia were detected by decoration for acetylated tubulin. Primary cilia on cultured mouse fibroblasts were first reported by Wheatley [18], and their cell cycle-dependent generation is well acknowledged [19,22,51]. Formation of primary cilia in vitro is commonly induced by cultivation under reduced serum concentration (so-called “serum starvation” conditions) for 48 h [22]. We observed that by using the exhausted medium supernatant of growing NIH3T3 cells, cilia induction in NIH3T3 cells (sub-line called here charge I) is at least as effective as in serum starved medium (Figure 1A). Decoration of primary cilia for axonemal tubulin acetylation is exemplified in Figure 1A. In standard medium with 10% FCS ~3% of cells are ciliated (*n* = 44 ciliated cells/1504 total cells). When serum starved (DMEM+ 0.5% FCS), ~7% of cells are ciliated corresponding to a twofold increase (*n* = 104/1409). Cultivation in the old, exhausted medium is even more effective resulting in an increase of ciliated cells to ~13% corresponding to a 4x increase (*n*= 181/1,432). The inductive capacity of the exhausted medium is most likely caused by the depletion of nutrients possibly assisted by factors secreted from NIH3T3 cells during cultivation.

An increase in ciliation was already observed even after 18 h of cultivation in exhausted/used medium (Figure 1B). In this case, a different NIH3T3 sub-cell line (named here charge II) was used having ~18% ciliated cells in the standard medium (*n* = 1563 cells counted) that increased to ~30% after 18 h cultivation in the exhausted medium (*n* = 3100). All experiments were repeated three times, and *n* is always the total number of counted cells.

### 3.2. Long-Term Cultivation Compromised Cytoplasmic and Axonemal Tubulin Acetylation

**Figure 2:**
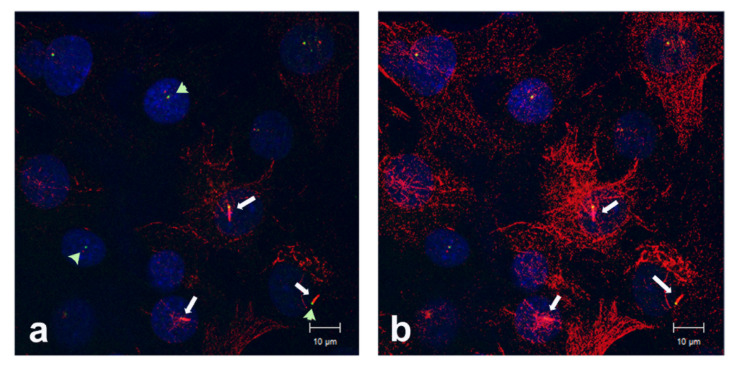
Tubulin acetylation and primary cilia in the NIH3T3 F sub-cell line. Cells were seeded at a density of 2 × 10^5^ cells per well, cultivated in normal medium for 24 h, followed by cultivation in exhausted/used medium for another 48 h. Cells were processed for immune cytology using the decoration of acetylated tubulin to identify primary cilia (red, arrows), and ODF2 to highlight the centrosomes and basal bodies (green, arrowheads). Different intensities in (**a**) and (**b**) to demonstrate detection of primary cilia even when cells are highly acetylated. Bars: 10 µm.

We noticed that different laboratory strains of NIH3T3 cells differ in their capacities to generate primary cilia with acetylated axonemal tubulin (see above, charge I and II, and Figure 1). In standard NIH3T3 culture medium, without enforced induction of cilia formation, the percentage of ciliated cells differ between ~3% and ~18% (Figure 1A,B). Even an increased cell density did not necessarily provoke a remarkable increase in ciliated cells when different cell charges were compared. Albeit 2 × 10^5^ cells of NIH3T3 cells of charge I were seeded per 6-well plate and cultivated for 3 days, only ~3% of cells are ciliated, whereas in NIH3T3 cells of charge II, seeded at 0.5 × 10^5^ cells per 6-well plate only and cultivated for just 18 h, ~18% of cells are ciliated in standard medium. Additionally, we repeatedly observed the variability in ciliation in the course of our studies, e.g., in a further NIH3T3 sub-cell line (named here NIH3T3 F). In normal conditions, cilia with an acetylated axoneme were found in ~6% of NIH3T3 F cells, whereas ciliation was increased to 29% in old medium (normal conditions: *n* = 2024, duplicate experiment; old medium: *n* = 474). Acetylation of cytoplasmic microtubules served as positive control, indicating that primary cilia, if present, should be decorated as well (Figure 2). Furthermore, primary cilia, when present, were easily identifiable (Figure 2).

Our results indicate that NIH3T3 sub-strains are highly variable in their ability to generate acetylated primary cilia. As the cells were cultivated at similar conditions, and equally treated for immune cytology afterwards, this observation suggested that the different ciliogenic capabilities are essentially not based on the culture conditions, but instead most likely reflect the intrinsic propensities of the cells in question. These results were nevertheless unexpected, awaiting further investigations.

**Figure 3:**
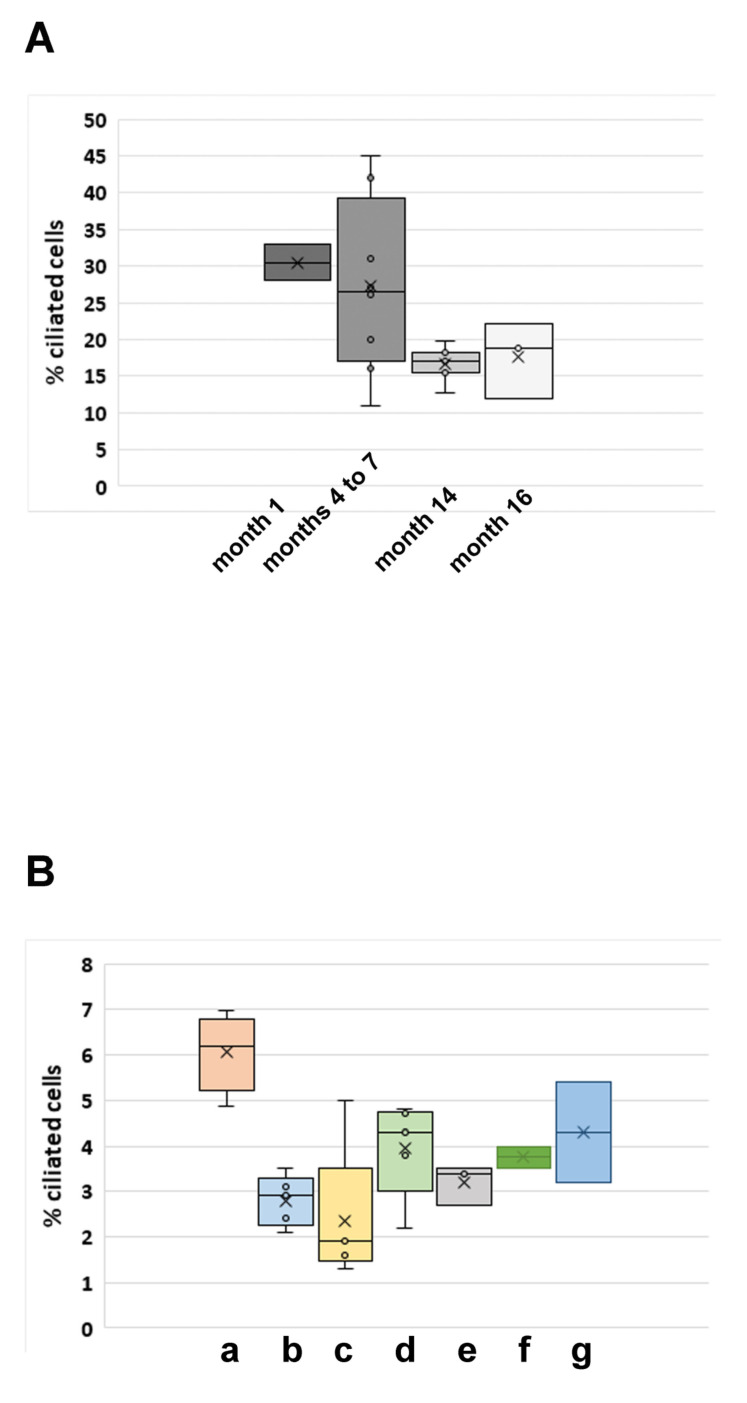
Decrease of acetylated cilia by long-term cultivation of NIH3T3 cells. (**A**) The NIH3T3 sub-cell line charge III was cultivated over a period of 16 months and routinely inspected for the presence of acetylated primary cilia. Cells were cultivated in standard medium (DMEM+10% FCS) at a density of 1 × 10^5^ cells per 6-well plate for 1 day, then immune-decorated for acetylated tubulin, and had their primary cilia counted. (**B**) Decrease of ciliation from ~6% to ~3% after approximately two years of intermittent cultivation of NIH3T3 sub-cell line F/F-con. Cells were cultivated in the standard medium at a density of 1.2–4.8 × 10^5^ cells per 6-well plate at the beginning of the experiment (a, *n* = 2037, four biological replicates), and later on at a density of 2.5 × 10^5^ cells per 6-well plate (b–g). Cells were either cultivated in standard medium for 24 h followed by immune-decoration (a, b, d, f, g) or were first grown in standard medium for 24 h followed by 48 h in serum starvation medium [DMEM + 0.5% FCS] before processing for immune decoration of acetylated tubulin (c, e). Inhibition of HDAC6 by TSA (d, e), of SIRT2 by nicotinamide (f), and of both HDAC6 and SIRT2 (g). (b: five biological replicates, *n* = 2463; c: five biological replicates, *n* = 2610; d: five biological replicates, *n* = 2522; e: three biological replicates, *n* = 1349; f: two biological replicates, *n* = 987; g: two biological replicates, *n* = 998). a: start of the experiment, standard medium (nm), b–g: after ~ two years of intermittent cultivation, b: nm, c: serum starvation medium (ssm), d: nm + TSA, e: ssm + TSA, f: nm + nicotinamide, g: nm + TSA + nicotinamide. The median is represented as a line, the average as a cross. Individual values are shown as a circle.

We first asked whether the ability to generate acetylated primary cilia might be affected by long-term cultivation, as all cell lines have routinely been used for some time, including repeated cycles of freezing–thawing for storage and re-culturing. We, therefore, retrospectively explored whether a change in ciliation has occurred over time in a specific NIH3T3 cells. The NIH3T3 sub-cell line of charge III has been constantly cultivated in the standard medium over ~16 months and primary cilia have been routinely inspected by immune decoration for acetylated tubulin. At the beginning of cultivation, ~30% of cells were ciliated, whereas approximately one year later the percentage of ciliated cells has dropped to ~17%, corresponding to a 50% decrease (1st month of cultivation: *n* = 1023, two biological replicas (2×); 4th to 7th months of cultivation: *n* > 1000, 9×; 14th months of cultivation: *n* = 3661, 7×; 16th months of cultivation: *n* = 781, 3×) (Figure 3A).

To verify the observation of a decline of acetylated primary cilia during cultivation, we repeated the experiment by using the NIH3T3 sub-cell line F that has ~6% acetylated primary cilia in standard medium when seeded at a cell density of 1.2–4.8 × 10^5^ per 6-well. Again, we found a similar reduction in primary cilia formation when cells were cultivated for a long time. Approximately two years after intermittent cultivation, ciliation dropped to ~3% when cultivated in the standard medium at a cell density of 2.5 × 10^5^ cells per 6-well plate corresponding to a decrease to ~50% (these cells are now named NIH3T3 F-con to discriminate them from the starting ones, NIH3T3 F, and to highlight their transformation). Even culturing in serum starvation medium did not result in a significant increase in ciliated cells (Figure 3B). As the decline in ciliated primary cilia might be caused by an increased deacetylase activity of HDAC6 or SIRT2, we inhibited their enzymatic activities by the addition of drugs. However, inhibiting the tubulin deacetylase HDAC6 by TSA did not result in a significant increase in ciliation, detected by the decoration of acetylated tubulin, neither in standard medium nor in serum starvation medium (Figure 3B(d,e)). Furthermore, inhibiting the tubulin deacetylase SIRT2 by nicotinamide, or both HDAC6 and SIRT2 simultaneously, did also not significantly increase acetylated cilia (Figure 3B(f,g)). The effectivity of the histone deacetylase inhibitors was confirmed several times by investigating ciliation in another NIH3T3 sub-cell line either simultaneously or independently, showing increased ciliation in the range of ~1.6-fold in the presence of nicotinamide and up to ~3-fold in the presence of both inhibitors (not shown).

**Figure 4:**
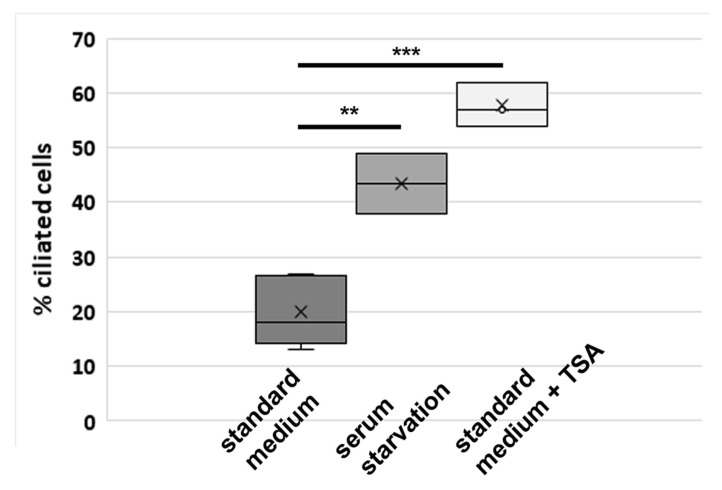
Increase of ciliation by either serum starvation or TSA-mediated inhibition of HDAC6 in the NIH3T3 sub-cell line “DSMZ”, which was freshly obtained from DSMZ. Cells were either cultivated in standard medium for 24 h (without or with the addition of TSA) or in serum starvation medium for another 48 h before processing for immune decoration for acetylated tubulin. Standard medium: five biological replicas, *n* = 1555; serum starvation: duplicate experiment, *n* = 1035; standard medium + TSA: triplicate experiment, *n* = 1362). Statistically significant differences of ciliation according to Student’s *t*-test: *p* ** standard medium versus serum starvation, *p* *** standard medium without TSA versus with TSA.

To further corroborate our results, we analyzed ciliation in a new NIH3T3 cell line freshly obtained from DSMZ (here called NIH3T3 “DSMZ”). Cells were either cultivated in standard medium, in serum starved medium, or in standard medium with the addition of the HDAC6 inhibitor TSA, and processed for decoration of acetylated tubulin. In standard medium, ~20% of cells are ciliated comprising an acetylated axoneme. Incubation in medium with reduced serum concentration for 48 h caused an increase in ciliation to ~44%, and cultivation in standard medium with 10% FCS for 24 h but in the presence of the HDAC6 inhibitor TSA provoked a further increase up to ~58% that corresponds to a 2.9-fold increase (Figure 4). Experiments were performed in replicates (2-fold up to fivefold), and revealed statistically significant differences of ciliation. According to Student’s *t-*test between standard medium and serum starvation medium (*p* **), and between standard medium without TSA versus with TSA (*p* ***).

**Figure 5:**
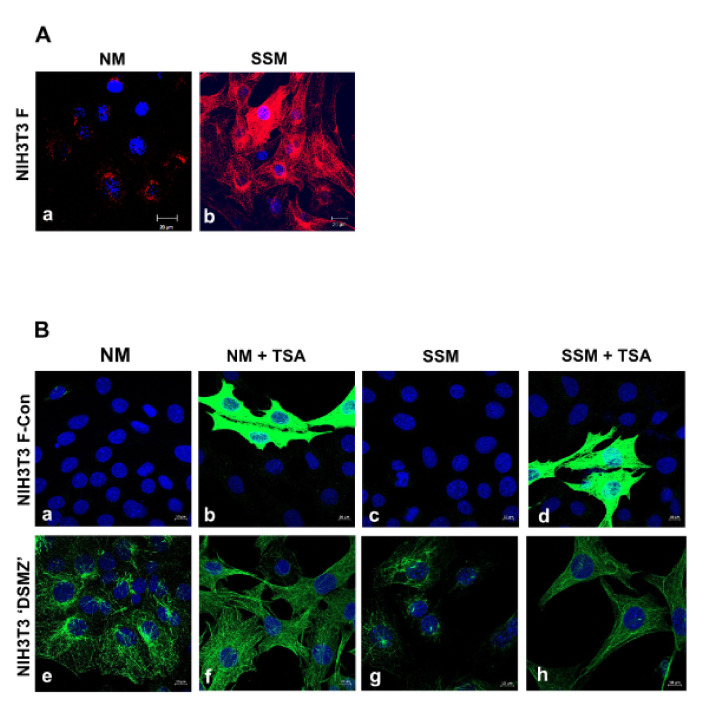
Acetylation of cytoplasmic tubulin in NIH3T3 F, NIH3T3 F-con, and NIH3T3 “DSMZ” sub-cell lines. (**A**) Cytoplasmic tubulin acetylation in NIH3T3 F cells increased in serum-starved medium. Cells were seeded at 2 × 10^5^ cells per 6-well plate in normal medium (NM) and cultivated for 24 h. Thereafter, the medium was exchanged for serum starvation medium (SSM), and cells cultivated a further 48 h. Immune decoration for acetylated α-tubulin (red). Nuclear counterstain with DAPI (blue). Bars: 20 µm. (**B**) Cytoplasmic tubulin acetylation in NIH3T3 F-con cells (derived from NIH3T3 F cells) and NIH3T3 “DSMZ” cells dependent on culture conditions and TSA-mediated HDAC6 inhibition. NIH3T3 sub-cell line F-con (**a**–**d**) and newly obtained NIH3T3 cells from DSMZ (**e**–**h**) were cultivated in standard medium (a,b,e,f; NM) or serum starvation medium (c,d,g,h; SSM) either in the absence of TSA (a,c,e,g) or in the presence of TSA (b,d,f,h; +TSA). To demonstrate even a low amount of cellular tubulin acetylation pictures in a to d were captured by overexposure. Decoration of acetylated tubulin in green, nuclear counterstain with DAPI in blue. Bars: 10µm.

Furthermore, when inspecting the cytoplasmic tubulin acetylation in the NIH3T3 F-con and the NIH3T3 “DSMZ” sub-cell lines, which responded differently to both, either serum starvation or HDAC6-inhibition, we observed that in NIH3T3 F-con cells acetylation of cytoplasmic tubulin is not visibly increased when cultivated in serum starvation medium, and only a few cells showed increased acetylation of cytoplasmic tubulin when HDAC6 is inhibited by TSA (Figure 5B, compare (a) to (c), and (b) to (d)). However, increased tubulin acetylation, albeit only in a few cells, confirmed the efficacy of HDAC6 inhibition by TSA. In contrast, NIH3T3 “DSMZ” cells are highly acetylated even in standard medium, and acetylation increased further by the addition of TSA (Figure 5B(e,f)), whereas increased acetylation in serum starved medium is hardly visible in these cells (Figure 5B(g,h)). Of note, the differences in cytoplasmic tubulin acetylation between NIH3T3 F and NIH3T3 F-con cells, which are derived from the former one, are evident especially in serum starvation conditions (Figure 5A(a,b)).

**Figure 6:**
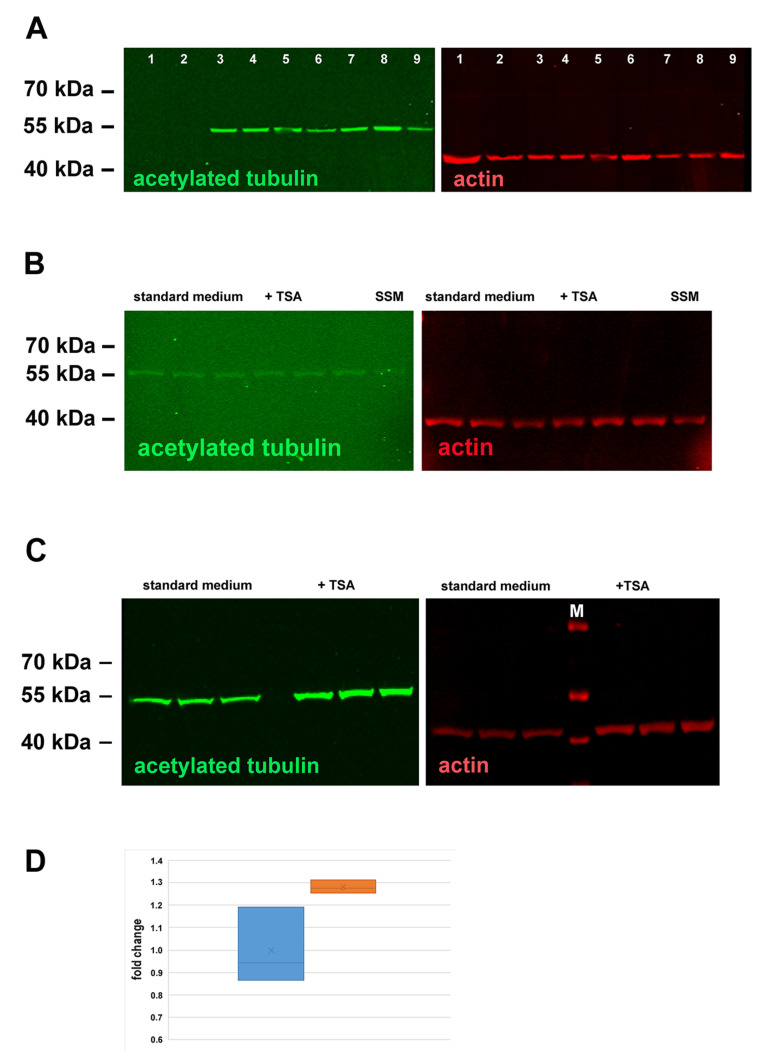
Tubulin acetylation in NIH3T3 F-con and “DSMZ” cells. (**A**) Serum starvation induced an increase in tubulin acetylation in NIH3T3 “DSMZ” but not in NIH3T3 F-con cells. Protein lysates equivalent to ~3 × 10^5^ cells were loaded per lane. 1: NIH3T3 F-con cultivated in standard medium; 2: NIH3T3 F-con cultivated in serum starvation medium; 3–5, 7, 8: NIH3T3 “DSMZ” cells cultivated in serum starvation medium; 6, 9: NIH3T3 “DSMZ” cells cultivated in standard medium (all different biological replicates). A reduced green fluorescence is obvious in lanes 6 and 9 albeit more proteins were loaded demonstrated by the increased red ß-actin fluorescence. (**B**) Inhibition of HDAC6 by TSA did not cause increased tubulin acetylation in NIH3T3 F-con cells. NIH3T3 F-con cells cultivated in standard medium (3 lanes), standard medium in the presence of TSA (+TSA, 3 lanes), or serum starvation medium (SSM, one lane). An equivalent of ~3 × 10^5^ cells was loaded per lane. (**C**) Inhibition of HDAC6 by TSA caused increased tubulin acetylation in NIH3T3 “DSMZ” cells. Cells were either cultivated in standard medium or in standard medium with TSA. Protein lysates equivalent to ~3 × 10^5^ cells per lane were loaded. Triplicate loadings. M: mass ruler. Fluorescent detection of antigens: ß-actin in red, acetylated tubulin in green. (**D**) HDAC6 inhibition by TSA induced increased tubulin acetylation. Acetylated tubulin and ß-actin from subfigure (**C**) were quantified from each lane and the relative quantity of acetylated tubulin calculated. The fold changes were calculated related to the mean relative quantity of acetylated tubulin in standard medium. Blue: relative quantity of acetylated tubulin in standard medium, red: relative quantity of acetylated tubulin in standard medium with TSA (Student’s *t*-test: *p* * 0.024).

Quantitative Western blotting corroborated the immune-fluorescent data showing that serum starvation conditions caused an increase in cytoplasmic tubulin acetylation as compared to cultivation in the normal medium when using the NIH3T3 “DSMZ” cell line that responded to these conditions by the formation of acetylated primary cilia (Figure 6A). However, in NIH3T3 F-con cells, in which generation of acetylated primary cilia was not induced by serum starvation, tubulin acetylation is hardly visible without any increase in the serum starved medium (Figure 6A(1,2)). Furthermore, inhibiting the histone deacetylase HDAC6 with TSA did not cause increased tubulin acetylation in NIH3T3 F-con cells (Figure 6B). In contrast, inhibition of HDAC6 by TSA resulted in increased tubulin acetylation in NIH3T3 “DSMZ” cells (Figure 6C). Although hardly detectable in the fluorescent image, quantizing of fluorescent images revealed a statistically significant increase in tubulin acetylation in the presence of TSA (according to Student’s *t*-test *p* *) (Figure 6D).

To sum up, our data show that long-term cultivation affected tubulin acetylation causing a decline in both cytoplasmic and axonemal tubulin acetylation. Furthermore, an increased activity of tubulin deacetylases, especially of the major tubulin deacetylase HDAC6, seems neither causative for the decline of cytoplasmic nor axonemal tubulin acetylation in NIH3T3 F-con cells, which were derived from NIH3T3 F cells by long-term cultivation. In this case, only a few cells showed increased acetylation of cytoplasmic tubulin when HDAC6 is inhibited by TSA (Figure 5B, compare (a) to (b), and (c) to (d)). In contrast, NIH3T3 “DSMZ” cells, which responded to serum starvation by an increase in acetylated cilia, responded to HDAC6-inhibition by an increase in tubulin acetylation and acetylated cilia already in standard medium (Figure 4 and Figure 6). Our results thus indicate a correlation between overall tubulin acetylation and the generation of acetylated primary cilia and, furthermore, between long-term cultivation and decreased tubulin acetylation, raising further questions about the drop of tubulin acetylation and the relationship between tubulin acetylation and the formation of primary cilia.

### 3.3. Reduced Tubulin Acetylation Is Caused by Repressed Atat1 Expression

**Figure 7:**
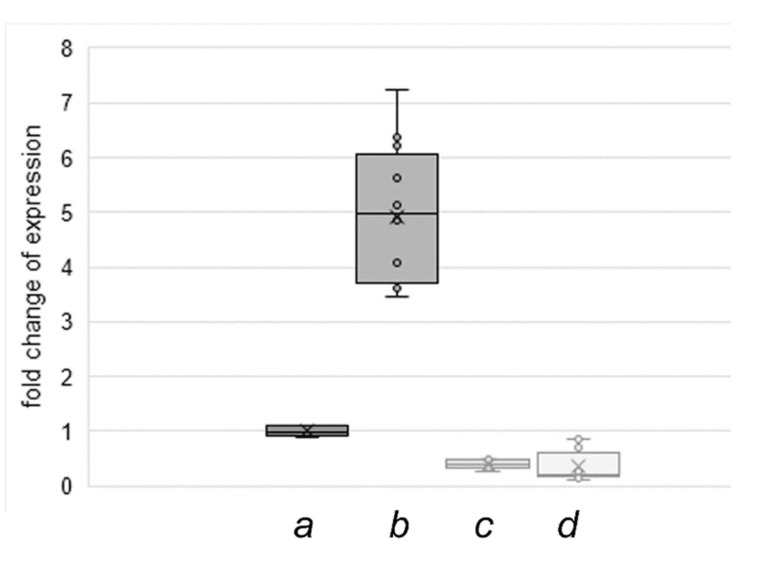
*Atat1* expression in standard medium and serum starvation medium in NIH3T3 “DSMZ” and NIH3T3 F-con cells. Serum deprivation induced *Atat1* expression in NIH3T3 “DSMZ” cells (b) compared to cultivation in standard medium (a). In NIH3T3 F-con cells *Atat1* expression is repressed in standard medium (c) and is also not induced by serum deprivation (d). Three to four biological replicates. Triplicate measurements. Student’s *t*-test all *p* *** related to NIH3T3 “DSMZ” cultivated in standard medium. The median is represented by a line, the average by a cross and individual values by circles.

Long-term cultivation of NIH3T3 cells provoked a decrease in tubulin acetylation and acetylated primary cilia, which is not caused by deacetylation due to enforced activity of the major tubulin-deacetylase HDAC6. Thus, a likely explanation is an impairment of the tubulin acetyltransferase ATAT1. We investigated the relative expression of *Atat1* in NIH3T3 F-con cells with low tubulin acetylation and in NIH3T3 “DSMZ” cells with inducible tubulin acetylation by qRT-PCR. Expression of *Atat1* was related to the expression of the housekeeping genes *Gapdh* and *Hprt.* The fold changes in expression of *Atat1* between the different sub-cell lines and culture conditions were calculated defining the relative expression of *Atat1* in the “DSMZ” sub-cell line (NIH3T3 “DMSZ”), cultivated in standard medium, as the ground state (=1). Cultivation of this sub-cell line in serum starvation medium for 48 h caused an approximately 6-fold increase in *Atat1* expression (Figure 7). These data are consistent with the observed increase of tubulin acetylation and primary cilia formation under these conditions (Figure 4, Figure 5 and Figure 6). However, in the NIH3T3 F-con sub-cell line *Atat1* expression is below that of NIH3T3 “DSMZ” cells when cultivated in either standard medium or serum starvation medium (Figure 7). *Atat1* expression is, therefore, not induced by serum deprivation in NIH3T3 F-con cells thus explaining the loss of tubulin acetylation. Thus, we conclude that the decline in tubulin acetylation by long-term cultivation is caused by impaired *Atat1* expression.

### 3.4. Induction of Primary Cilia Formation Irrespective of Tubulin Acetylation

**Figure 8:**
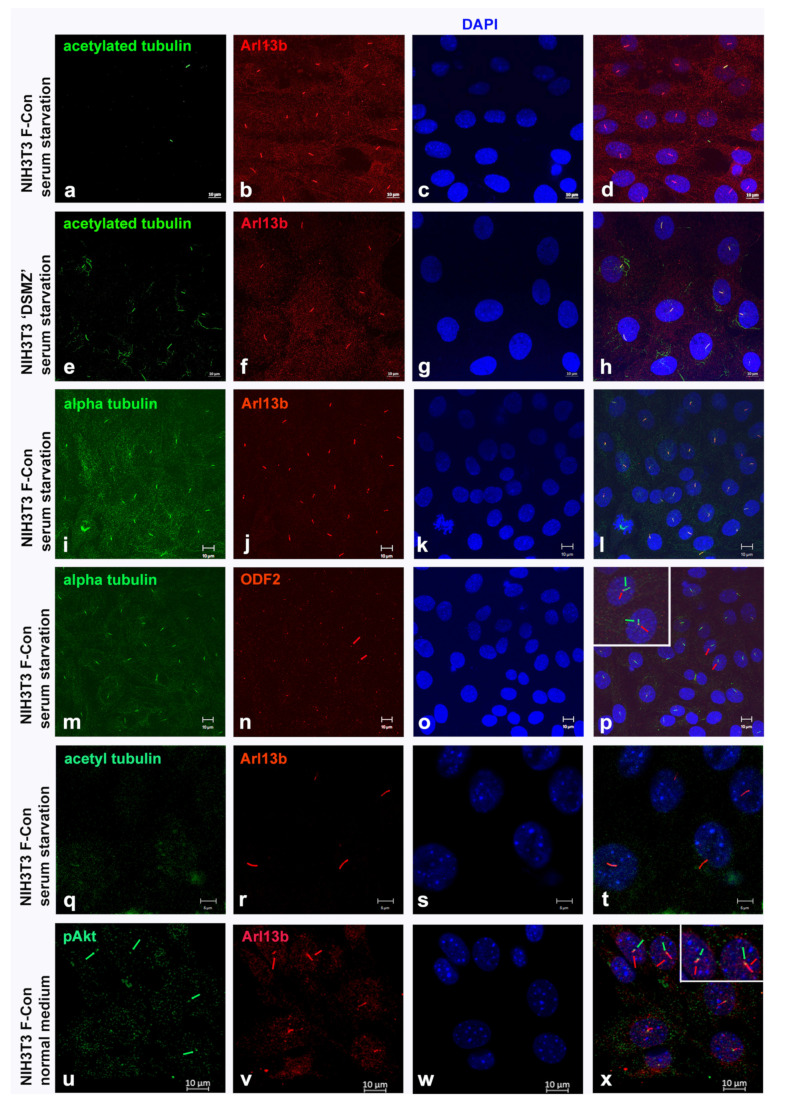
Arl13b is a reliable marker for primary cilia. In the NIH3T3 sub-cell line, F-con only a few of the primary cilia detected by Arl13b-staining are also decorated for acetylated tubulin (**a**–**d**), whereas all primary cilia in the newly obtained sub-cell line from DSMZ (NIH3T3 “DSMZ”) contain both, acetylated tubulin and Arl13b (**e**–**h**). Arl13b-positive structures in NIH3T3 F-con comprise an axoneme, by detection of α-tubulin (**i**–**l**), and a basal body, decorated by ODF2 (**m**–**p**; pink arrows depict ODF2-decorated basal bodies highlighted in red in (**n**,**p**). Enlargement of two cells with primary cilia in subfigure (**p**).). Primary cilia in NIH3T3 F-con cells are functional signal transducers by demonstrating the activated AKT-kinase (pAKT) at the basal bodies (**u**–**x**; pAKT signals at the base of the primary cilia are decorated in green and are depicted by light green arrows in u and x. The primary cilia are stained in red and are depicted by pink arrows in v and x. Enlargement of two cells with primary cilia in (**x**)). Even basal bodies are not enriched for acetylated tubulin in NIH3T3 F-con (**q**–**t**). Bars: 5 µm (**q**–**t**), and 10 µm (all other pictures).

**Figure 9:**
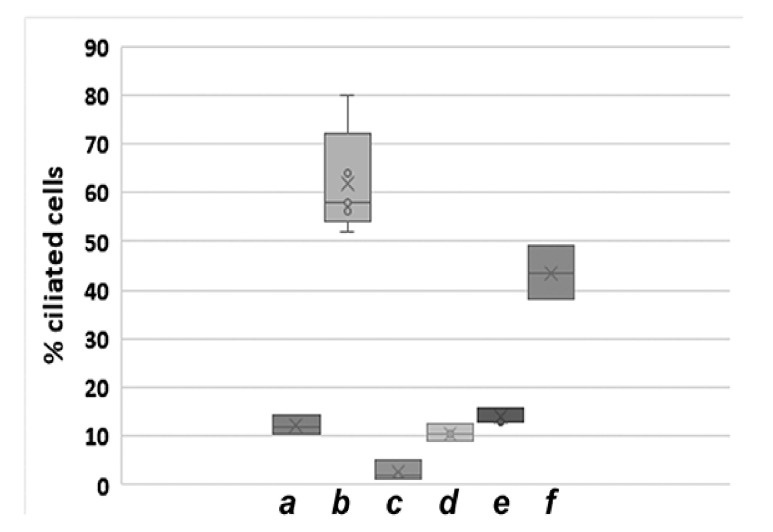
Arl13b is a reliable marker for primary cilia. Induction of ciliation by serum deprivation in NIH3T3 “DSMZ” as well as NIH3T3 F-con cells. NIH3T3 sub-cell line F-con (a–d) and NIH3T3 “DSMZ” cells (e,f) were cultivated in standard medium (a,e), serum starvation medium (b,c,f), or standard medium with TSA (d). Using Arl13b immune staining, primary cilia increased from 12.2% in standard medium (a, *n* = 2297) to 62% when serum starved for 48 h (b, *n* = 2627) in sub-cell line F-con, whereas less than 3% primary cilia were detected by acetylated tubulin decoration in serum starved cells (c, *n* = 1580). Inhibition of HDAC6 by TSA did not cause an increase in Arl13b-decorated cilia in standard medium (d, ~10%, *n* = 1535). In the NIH3T3 “DSMZ” cell line Arl13b-decorated primary cilia increased from 14% in standard medium (e, *n* = 1555) to ~44% in serum starvation medium (f, *n* = 1035). Serum starvation of “DSMZ” cells two biological replicates, all other three biological replicates.

Our results indicate that long-term cultivation compromised acetylation of tubulin in primary cilia and the cytoplasm and that increased activity of HDAC6 is not causative. We, therefore, asked whether tubulin acetylation is the reliable marker for cilia or if they might have been overlooked. To this end, we investigated the formation of primary cilia using decoration for both acetylated α-tubulin and Arl13b, simultaneously. In the NIH3T3 “DSMZ” sub-cell line, every primary cilium is stained for both, acetylated α-tubulin and Arl13b (Figure 8e–h). However, in the NIH3T3 sub-cell line F-con we found less than 3% acetylated tubulin-positive primary cilia, but 62% Arl13b-positive structures when cultivated in serum starvation medium (Figure 9b,c). Thus, only approximately every twentieth primary cilium was detected when decorated for acetylated α-tubulin (Figure 8a–d). Furthermore, even the NIH3T3 F-con cells responded to serum deprivation by an increase in ciliation. Decoration for α-tubulin demonstrated the axoneme in Arl13b-positive structures in the F-con sub-cell line proving that they are indeed primary cilia (Figure 8i–l). Furthermore, the detection of the basal body marker ODF2 at the base of the α-tubulin-containing axonemata corroborated their identification as primary cilia (Figure 8m–p). Moreover, we demonstrated that all primary cilia are functional organelles by detection of phosphorylated AKT (pAKT) at the basal body in all Arl13b-positive cilia (Figure 8u–x). These results demonstrate that tubulin acetylation is dispensable for the formation of functional primary cilia, and even basal bodies were not enriched for acetylated tubulin (Figure 8q–t). Furthermore, serum deprivation induced quiescence and in turn ciliation even in cells with low tubulin acetylation.

### 3.5. Correlation between Low Atat1 Expression and Increased Proliferation

**Figure 10:**
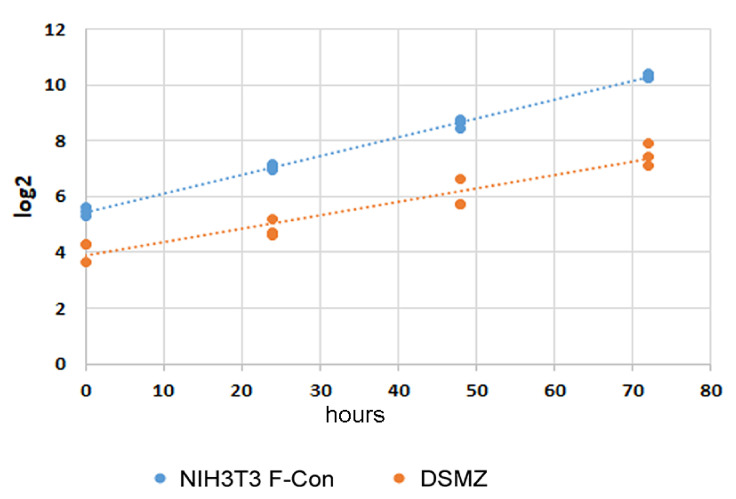
Loss of Atat1 expression correlates with increased proliferation rate. NIH3T3 sub-cell lines “DSMZ” and F-con were seeded in 12-well plates and cultivated in standard medium. At the indicated time points, cells were fixed and stained with DAPI. 20 pictures from each well were taken and the nuclei counted automatically. The growth curves were depicted as log2. Triplicate experiments.

Our results show that long-term cultivation of fibroblast cells caused a decline in *Atat1* expression and tubulin acetylation. Primary cilia are nevertheless generated, albeit most of them are without detectable tubulin acetylation. Furthermore, cells respond to serum deprivation by cell cycle exit and the formation of Arl13b-positive primary cilia. However, the “transformation” of the cell population from cells with detectable tubulin acetylation at the beginning into those with a strong decline in tubulin acetylation and *Atat1* expression after long-term cultivation opens the question if the decline of *Atat1* expression provides a selective advantage to the affected cells that, eventually, let overgrow and substitute less fit cells of the population. To this end, we performed proliferation assays using the “DSMZ” and the NIH3T3 F-con sub-cell lines. Cells were seeded at identical densities into 12-well plates and the cells fixed and counted at regular intervals using always triplicate experiments. The results demonstrate that NIH3T3 F-con cells have a higher proliferation rate than NIH3T3 “DSMZ” cells (Figure 10). Whereas NIH3T3 F-con cells have a doubling time of 14.9 h in normal medium, NIH3T3 “DSMZ” cells have a doubling time of 21 h under the same conditions. Our results, therefore, demonstrate the correlation between an increased proliferation rate and the decline/loss of *Atat1* expression indicating that cells without ATAT1 and tubulin acetylation have a selection advantage eventually resulting in the substitution for those cells that are less fit because of *Atat1* expression.

## 4. Discussion

Fibroblasts are essential cellular components of the connective tissue of vertebrates and play fundamental roles in diverse biological processes as wound healing, inflammation, and cancer. Like most cells of the vertebrate body, the fibroblast harbors a primary cilium as well. Primary cilia are sensory organelles transmitting mechanical and chemical cues to the cell center. In fibroblasts, they are essential for the directed migration and proliferation as a response to injury and are therefore crucial for wound healing. Although cell cycle exit is the main trigger for cilia formation, the environmental cues leading to induction of primary cilia are not fully understood.

We used here the established mouse embryonic fibroblast cell line NIH3T3 as a model to investigate the induction of primary cilia. Using acetylated α-tubulin as the well-accepted marker for primary cilia, we observed a remarkable difference in ciliation in diverse sub-strains of NIH3T3 cells. These differences are already found when cultivated in standard medium. Furthermore, not all sub-cell lines reacted to serum starvation with the induction of primary cilia. Of note, only few cells of the NIH3T3 F-con sub-cell line have a primary cilium when detected by tubulin acetylation, but the cell line is indeed highly ciliated when using the ciliary marker Arl13b for decoration indicating that its ciliation capacity is not significantly altered. Instead, the reduction of acetylated primary cilia is accompanied by an overall reduction of tubulin acetylation that is not caused by the increased activity of the major tubulin deacetylase, HDAC6. Furthermore, cells that responded to serum deprivation by an increase of tubulin acetylation and primary cilia formation are transformed into non-responders showing no increase of tubulin acetylation when cultivated for a long time. Besides that, we did not observe any remarkable change in proliferation, morphology, or heterochromatic foci indicative of cellular senescence [58,59]. As the HDAC6 activity is not responsible for the decline, we asked whether there is an altered expression of the major α-tubulin acetyltransferase, *Atat1*. We observed that NIH3T3 F-con cells, which are marginally acetylated, have a very low expression of *Atat1* in both culture conditions and that even known cilia-inducing conditions are ineffective. The reduced expression of *Atat1* may be caused either by inhibition or silencing of the *Atat1* gene putatively induced by the culture conditions since whole genomic PCR revealed the presence of the *Atat1* gene (not shown).

A conceivable explanation for the aforementioned transformation of cells by long-term cultivation is cell competition and natural selection as also observed by Mamada et al. [60]. In our case, NIH3T3 cells with reduced *Atat1* expression and tubulin acetylation may have a growth advantage over cells with high *Atat1* expression and increased tubulin acetylation, irrespective if tubulin acetylation is just a visible marker or is causative. Thus, the favored cells will overgrow and displace the other cells. As ATAT1 controls cell growth via inhibition of the AKT kinase, the reduced expression of *Atat1* in NIH3T3 F-con cells indicates a growth advantage, which we confirmed by the proliferation assays [50]. A slightly increased proliferation rate has also been observed in *Atat1^-/-^* MEFs, thus supporting our results [43]. Furthermore, we demonstrated the activation of the PI3K signaling cascade in these cells, with low *Atat1* expression, by identification of the active AKT kinase (pAKT) at the basal bodies. As opposed to NIH3T3 “DSMZ” cells, NIH3T3 F-con cells have been cultivated for a long time typically experiencing periodic cycling from semi-used medium back into fresh medium. These cycling conditions might provide a selective pressure that eventually caused the transformation of the cell population. Besides the observed differences in *Atat1* expression and tubulin acetylation, the two cell lines may differ in additional features that contribute to their different proliferation. However, when culturing in serum-deprived medium, both cell lines stop proliferation (not shown.

Although we found that NIH3T3 F-con cells proliferate faster than NIH3T3 “DSMZ” cells a significant difference in the percentage of their ciliated cells were not found. In NIH3T3 F-con ~12% of cells harbor an Arl13b-positive primary cilium, whereas ~14% were found in NIH3T3 “DSMZ” cells. Thus, it seems that an impact of primary cilia on the cell cycle is unlikely.

Even though we observed a decline in tubulin acetylation and a decrease in acetylated primary cilia in long-term cultured fibroblasts, a high percentage of cells is nevertheless ciliated when using Arl13b for their decoration. Arl13b (ADP-ribosylation factor-like protein 13b) is a small GTPase that associates with the ciliary membrane by N-terminal palmitoylation [61]. Arl13b is required to control the length of the microtubule-based, ciliary axoneme most likely by controlling protein trafficking to or from the cilium. Furthermore, Arl13b binds tubulin and seems to regulate tubulin modifications within the cilium, i.e., acetylation of tubulin is reduced in Arl13b^hnn^ mutant cells [62,63]. Our results, however, show that the presence of Arl13b in the cilium is not sufficient to promote tubulin acetylation, and primary cilia are functional without tubulin acetylation. Thus, Arl13b might promote modification of tubulin, as outlined by Larkins et al. [62]; however, in light of the observation that functional cilia are generated even in the absence of tubulin acetylation, this might be a secondary effect of Arl13b. It is conceivable that Arl13b, by interaction with axonemal tubulin, alters the tubulin lattice to enable access of ATAT1 for acetylation. Acetylated tubulin characterizes long-lived and stable MTs being either just a marker of microtubule age or protecting against mechanical stress and aging [38]. We found that all Arl13b-positive cilia are functional by detecting the active AKT kinase at their basal bodies and that means that axonemal tubulin acetylation is not essential for their functionality. Whether both kinds of primary cilia, either acetylated or not acetylated, differ either in stability or in age has not been investigated explicitly. Indications for a protective function of tubulin acetylation were not found. Additionally, it is hardly imaginable that there is an age difference between acetylated and non-acetylated cilia considering their cell cycle-dependent assembly/disassembly.

To sum up, we have demonstrated for the first time that reduced *Atat1* expression and tubulin acetylation correlate with increased proliferation and thus, most likely, provide a selective advantage in cell competition. As reduced tubulin acetylation and increased migratory behavior are hallmarks of epithelial-to-mesenchymal transition (EMT), in tumor formation as well as in wound healing, repression or silencing of *Atat1* might be considered as being essentially involved in the transformation process [37]. Furthermore, our observation has major implications on the interpretation of cell culture-based approaches. It is mandatory to consider the possibility that a cell line has been transformed by culture conditions over time causing an alteration of specific features.

## 5. Conclusions

Our results demonstrate a significant impact of long-term cultivation conditions on intrinsic cellular features. Of note, we observed a transformation towards cells with low *Atat1* expression and tubulin acetylation that correlated with a decrease of acetylated primary cilia. Tubulin acetylation is, therefore, not a robust and reliable ciliary marker. Instead, evaluation of ciliation demands the simultaneous usage of different ciliary markers. Furthermore, we provide evidence that cells with reduced *Atat1* expression and tubulin acetylation have a growth advantage resulting in overgrowth and eventually displacement of all other cells in the population. As the transition of epithelial cells into mesenchymal cells (EMT) in both tumor formation and wound healing is characterized by an increased cell migration as well as reduced tubulin acetylation, repression or silencing of *Atat1* might be crucially involved in the transition process. Additionally, these cells may have also increased proliferation rates.

## Data Availability

The datasets generated during the current study are available from the corresponding author on reasonable request.
